# Is there any difference between the distances created by towel clamp lifting and towel clamp plus manual lifting of the anterior abdominal wall for direct trocar entry in laparoscopic gynecologic surgery? A prospective interventional study

**DOI:** 10.4274/jtgga.2016.0203

**Published:** 2017-12-15

**Authors:** Taner A. Usta, Tolga Karacan, Evrim Ebru Kovalak, Ulviye Hanlı, M. Murat Naki

**Affiliations:** 1 Clinic of Obstetrics and Gynecology, Bağcılar Training and Research Hospital, İstanbul, Turkey; 2 Department of Obstetrics and Gynecology, Acıbadem University Faculty of Medicine, İstanbul, Turkey

**Keywords:** Abdominal wall lifting, laparoscopic entry, abdominal wall elevation

## Abstract

**Objective:**

Most surgeons prefer to perform anterior abdominal wall lifting during abdominal entry to avoid damage to intestines or main vessels. Anterior abdominal wall lifting is assumed to prevent vital organ injuries by creating an adequate distance prior to entry into the peritoneal cavity. In this study, we compared the distance created for trocar entry into the peritoneal cavity with towel clamp lifting and towel clamp plus manual elevation of the anterior abdominal wall.

**Material and Methods:**

Forty patients who underwent various laparoscopic procedures were enrolled. The study was performed in two steps: first the anterior abdominal wall was lifted using towel clamps (TC group), next the anterior abdominal wall was lifted via maximal manual elevation from the lower abdomen in addition to towel clamps (TCM group). The insertion distance of a plastic ruler into the abdomen was measured from the parietal peritoneum to the intra-abdominal structure in both groups.

**Results:**

There was a statistically significant difference between the two groups (TC group 3.9±1.5 cm vs. TCM group 4.5±1.5 cm, p<0.001). Correlation analysis of the relationship of distance with BMI in the study groups revealed a strong negative linear correlation [TC group vs. body mass index (BMI); r=-0.719, p<0.001 and TCM group vs. BMI, r=-0.749, p<0.001]. Correlation analysis of the relationship between the study groups and parity number revealed a weak negative linear correlation (TC group vs. parity number, r=-0.071, p=0.76 and the TCM group vs. parity number, p=0.61), which did not reach statistical significance.

**Conclusion:**

The recruitment of both towel clamps and manual elevation in anterior abdominal wall lifting provides significantly greater distance for trocar entry in laparoscopic surgery.

## INTRODUCTION

Complications that arise during abdominal cavity entry constitute about 50% of all complications encountered in laparoscopic surgery. Most surgeons prefer to perform anterior abdominal wall lifting during abdominal entry to avoid damage to the intestines or main vessels. Veress needles, direct trocar insertion, the Hasson technique, and visual trocar systems might be used for abdominal entry to create pneumoperitoneum. The conventional method of creating pneumoperitoneum in closed entry techniques entails blindly advancing the Veress needle or trocar from the umbilicus into the peritoneal cavity during abdominal wall lifting. Abdominal wall lifting might be performed manually and/or with the help of towel clamps (TC). The main goal of the procedure is to avoid intestinal and vascular injuries, and increase skin resistance to facilitate subcutaneous tissue perforation during abdominal entry (1,2).

Anterior abdominal wall lifting is assumed to prevent vital organ injuries by creating an adequate distance prior to entry into the peritoneal cavity. The primary objective of our study was to compare the distances created in towel clamp lifting, and towel clamp plus manual lifting of the anterior abdominal wall. Hence, we aimed to determine whether the additional manual upwards lifting of the anterior abdominal wall prior to laparoscopic entry provided any significant increase in the distance compared with the use of towel clamps alone. Furthermore, we compared the relationship of both procedures with body mass index (BMI) and parity number.

## MATERIAL AND METHODS

### Study design

The study was designed as a prospective observational clinical study and performed at Bağcılar Training and Research Hospital. Forty patients, who underwent laparoscopic surgery for benign and premalignant-malignant gynecologic diseases at the Clinic of Obstetrics and Gynecology between November 2013 and December 2015, were included in the study. The study did not alter the type or form of the planned surgical procedure. The study group consisted of volunteer patients. Patients were informed in detail about the entire scope of surgical procedure as well as all potential intraoperative and postoperative complications. Age, obstetric and gynecologic history, and family history of the patients were recorded in the first preoperative visit. Height and weight were measured to calculate BMI (kg/m^2^) preoperatively. All study procedures were performed in accordance with the Declaration of Helsinki. Institutional ethics committee approval was obtained, and written informed consent was obtained from each subject prior to the performance of any study procedures. The clinical trial registration number is NCT01726231 (ClinicalTrials.gov).

The required sample size was calculated, anticipating a goal of 25% increase in the abdominal wall to the nearest intra-abdominal structure distance, with a power value of 80% and an alpha-error value of 0.05. The number of subjects needed for the study was found as 36; 46 patients were enrolled in both groups. The 3 patients who refused to participate in the study at the last minute despite agreeing to undergo laparoscopy comprised 2 patients with severe adhesions (grade 4) (3) that precluded intra-abdominal imaging, and 1 patient with minimal umbilical herniation noted immediately before direct trocar entry; these patients were excluded from the study. In total, 40 patients were included in the study.

Antithrombotic prophylaxis was performed in line with the recommendations of the American College of Obstetrics and Gynecology and the American College of Chest Physicians to include early mobilization in patients at low risk, the use of gradual compression socks, and administration of 40 mg enoxaparin preoperatively 2 hours before the procedure and postoperatively until discharge in patients at moderate risk, and the use of gradual compression socks and administration of 40 mg enoxaparin preoperatively 2 hours before the procedure and postoperatively until week 4 in patients at high risk (4,5). Additionally, intermittent pneumatic compression devices were applied to both legs during the operation. All laparoscopic procedures were performed under general anesthesia. Gastric decompression was performed using oral-gastric tubes. Foley catheters were inserted preoperatively and removed 8 hours postoperatively. All study procedures were performed by 2 experienced surgeons.

### Study setting

Intra-umbilical incisions were performed in the supine position in all patients. Subcutaneous tissue thickness beneath the umbilicus was measured in centimeters using a plastic ruler prior to creating pneumoperitoneum. Next, the anterior abdominal wall was lifted vertically upwards using towel clamps on both sides of the umbilicus and manual lifting from the lower abdomen. A 10-mm disposable trocar was advanced into the abdomen at steady pressure under the guidance of index finger. The abdominal viscera were observed by introducing the telescope via the first trocar. The abdomen was inflated with CO2 gas to reach an intraperitoneal pressure of 20 mm Hg. A second 10-mm disposable trocar was inserted in the right lower quadrant in the Trendelenburg position. Examinations were performed via direct observation through the telescope in the second trocar.

**Figure 1:**
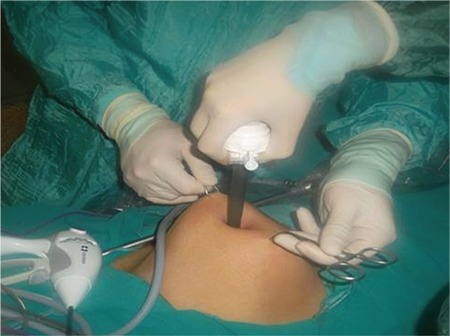
The skin was held and lifted using 2 towel clamps placed laterally at the level of the umbilicus, while the patient lay in the supine position

**Figure 2:**
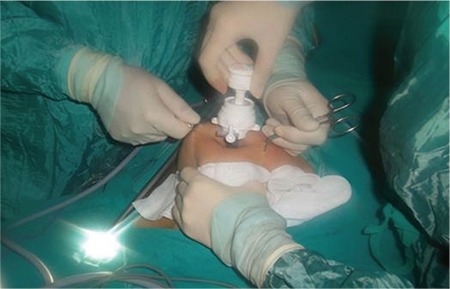
Manual lifting of the anterior abdominal wall from lower abdomen in addition to the use of 2 towel clamps at the level of the umbilicus, while the patient lay in supine position

The study was performed in two steps: first the anterior abdominal wall was lifted using towel clamps inserted on both sides of the umbilicus (TC group; Figure 1), next the anterior abdominal wall was lifted via maximal manual lifting from the lower abdomen in addition to towel clamps (TCM group; Figure 2). Distance obtained through anterior abdominal wall lifting was defined as the distance from the parietal peritoneum beneath the umbilicus to the closest intra-abdominal structure identified by direct observation.

**Figure 3:**
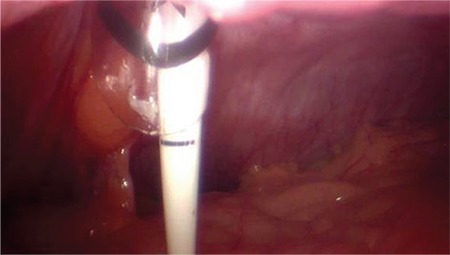
Image of the plastic ruler and demonstration of how to measure the distance from the anterior abdominal wall to intraperitoneal structures during anterior abdominal wall elevation while patient lies in the supine position. The plastic ruler is advanced through the umbilicus into the abdominal cavity and held at a 90-degree angle with its tip touching the closest visceral organ, under camera observation through the assistant trocar. The distance between the parietal peritoneum on the anterior abdominal wall and visceral organs are measured in centimeters

**Step 1 (TC group):** The telescope was removed to carry out the study procedures and the intra-abdominal CO2 gas was evacuated. The anterior abdominal wall was lifted using towel clamps on both sides of the umbilicus. Next, the telescope was re-inserted into the abdomen through a 10-mm assistant-trocar. A plastic ruler was advanced into the abdomen through the trocar at the umbilicus. The tip of the plastic ruler was held touching the closest visceral organ at a 90-degree angle under direct observation. The insertion distance of the plastic ruler into the abdomen was measured from the parietal peritoneum to the intra-abdominal structure in centimeters and recorded (Figure 3).

**Step 2 (TCM group):** In the second stage of the study, the lower abdomen was grasped manually midway between the umbilicus and the pubic symphysis and lifted, in addition to the towel clamps inserted on both sides of umbilicus (Figure 3). Next, the same measurement technique was used to determine how much the abdomen was lifted in centimeters.

All study data were analyzed using SPSS® version 19.0 (SPSS Inc., demo, Chicago, IL, USA). Descriptive statistics including numbers, percentages, means and standard deviation were used to interpret the data. The distribution of continuous variables was assessed using the Shapiro-Wilk test. Wilcoxon’s signed-rank test was used to compare data with non-uniform distribution. The relationship between continuous variables was analyzed using Spearman’s correlation analysis. The results were interpreted at 95% confidence intervals, and p<0.05 was interpreted as significant.

## RESULTS

A total of 40 females were included in the study. Demographics and baseline characteristics of the study patients are summarized in Table 1. The mean baseline subcutaneous tissue thickness at the umbilicus level was 3.3±1.0 cm (range, 1.2-6 cm) in the study population. There was a weak positive correlation between subcutaneous tissue thickness beneath the umbilicus and BMI (r=0.286); however, this relation did not reach statistical significance (p=0.08).

**Figure 4:**
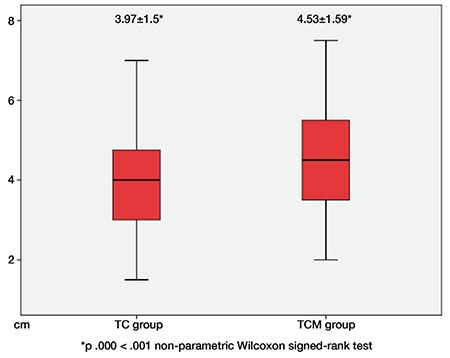
There was a statistically significant difference between the two groups TC: Towel clamps

The mean distance achieved during anterior abdominal wall lifting was 3.9±1.5 cm (range, 1.5-7 cm) in the TC group, and 4.5±1.5 cm (range, 2-7.5 cm) in the TCM group. There was a statistically significant difference between the two groups (p<0.05) (Figure 4).

Entry into the peritoneal cavity was achieved in the first trial in all patients. A minimal omental injury occurred in one patient during direct trocar entrance, whereby bleeding stopped spontaneously and did not preclude inclusion in the study.

**Figure 5:**
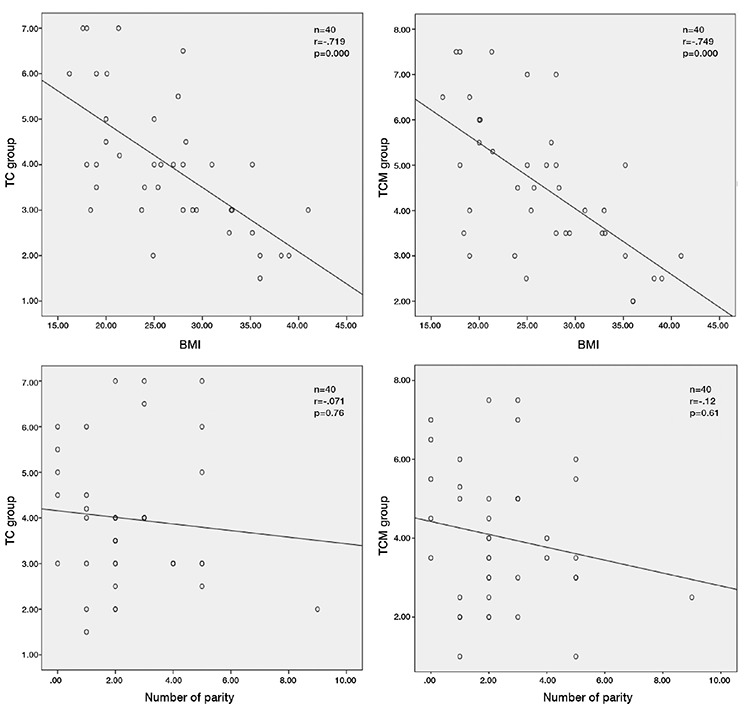
Correlation analysis of the relationship of distance with body mass index and parity number in the study groups BMI: Body mass index, TC: Towel clamps

Correlation analysis of the relationship of distance with BMI in the study groups revealed a strong negative linear correlation (TC group vs. BMI, r=-0.719, p<0.001; and TCM group vs. BMI, r=-0.749, p<0.001, Figure 5). The relationship with BMI had statistical significance in both groups.

Correlation analysis of the relationship between the study groups and parity number revealed a weak negative correlation (TC group vs. parity number, r=-0.071, p=0.76; and TCM group vs. number of parity, r=-0.12, p=0.61, Figure 5), which did not reach statistical significance.

## DISCUSSION

Creating pneumoperitoneum constitutes the primary and most important step of laparoscopic surgery. Four basic abdominal entry techniques have been described for this purpose: the classical Veress needle introduction, open (Hasson) entry technique, direct trocar insertion, and the visual trocar system. However, there is no consensus on the most effective and safest abdominal entry technique (2,6). The incidence of major vascular injuries and intestinal injuries has been reported as 0.02-0.5% and 0.06-0.1%, respectively (7,8). Among the vascular structures, the abdominal aorta, inferior vena cava, and iliac vessels are injured most commonly. The majority of major vessel injuries have been reported in patients with previous history of abdominal surgery and associated intra-abdominal adhesions. However, timely diagnosis of intestinal injury deserves special attention because laparoscopy-related mortality is more common in cases of missed intestinal perforations than major retroperitoneal vessel injuries (9,10).

Six principles should be followed to prevent injury to vital structures during entry into the peritoneal cavity: visualization, stabilization, adequate incision, controlled penetration, proper direction, and minimization of insertion (11). In clinical practice, the anterior abdominal wall is lifted upwards using towel clamps or manually from the lower abdomen. The objective of abdominal lifting is to distance the anterior abdominal wall from the intestines and vascular structures as much as possible before entry into the peritoneal cavity. Blind entry into the peritoneal cavity has been considered to be safer and more effective in this process (6,12).

No major complications other than a minimal omental injury were encountered in our study. In this technique, the trocar is inserted directly downwards at 90 degrees to the abdominal wall following upwards lifting of the anterior abdominal wall using towel clamps, as well as manual lifting from lower abdomen. Hence, a safe distance is established to prevent injury to the closest vital organ and risk of entry failure (e.g. extraperitoneal insufflation) is minimized. It appears that manual lifting of the anterior abdominal wall in addition to the use of towel clamps increases the success rate of direct trocar entry and facilitates entry.

The umbilicus is the most common site of entry into the abdominal cavity in laparoscopic surgery due to its unique characteristic features including being the thinnest and least vascular site of the anterior abdominal wall. The abdominal wall layers are at their thinnest at this level. This site is minimally influenced by body type and BMI (6). The presence of a thick abdominal wall might decrease tactile sensation and therefore complicate closed entry into the peritoneal cavity (via direct trocar or Veress needle) in obese patients. On the other hand, the abdominal wall lies too close to the retroperitoneal structures in thin patients (13). The main goal of the first entry in laparoscopic surgery is to prevent injury to vital structures in thin patients and reduce failed entry rates in obese patients (12).

Approaching the abdominal wall at a 90-degree angle is of primary importance for the success of entry into the peritoneal cavity in obese patients. Otherwise, the trocar or Veress needle might proceed in subcutaneous tissue and extraperitoneal insufflation risk might emerge. Classic texts recommended horizontal entry with the recruitment of 45-degree angle to prevent vascular injuries in thin patients; however, this has also been reported to potentially increase the risk of extraperitoneal insufflation (12,13). In our study, we found that vertical entry into the peritoneal cavity was feasible in thin patients (within the TCM group). The risk of injury to vital structures might be minimized with the recruitment of maximum abdominal wall lifting and controlled trocar entry at stable strength. Naturally, controlling entry axial force is easier for operators with stronger upper bodies and might pose a problem for female surgeons (10).

Lifting the fascia is technically easier in patients with low BMI. In our study, we noted that the distance between anterior abdominal wall parietal peritoneum and visceral organs decreased statistically significantly with increased BMI in both groups. However, this reduction was more prominent in the TC group. Our results also demonstrated that additional manual lifting performed in the TCM group provided greater lifting of the parietal peritoneum in patients with higher BMI. Previous studies have reported that the distance between the parietal peritoneum and closest intestinal organs increased only at the lifted parts of the abdominal wall, and blind abdominal entry had to be performed at a 90-degree angle (2).

Observation through the assistant trocar showed that maximal lifting of the anterior abdominal wall occurred only at the exact site of lifting in the TC group. Intestinal structures at the site of lifting might potentially get stuck in the anterior parietal peritoneum during abdominal lifting due to the cone-shaped configuration of the peritoneum at the base of the umbilicus (10,14). However, we saw that peritoneal tenting was removed with additional manual lifting of the anterior abdominal wall in the TCM group, providing the laparoscopist adequate distance in the direction of trocar in thin patients.

To the best of our knowledge, our study is the first to investigate the relationship between anterior abdominal wall lifting and parity number. Multiparity is currently the best known etiologic cause of rectus diastasis (15). The results of our study demonstrated a weak negative, though statistically not significant, correlation between parity number and the intra-abdominal distance created by anterior abdominal wall lifting. A possible explanation is the disruption of the integrity of thin, single-layer stratum consisting of skin, fascia, and peritoneum beneath the umbilicus due to multiparity (14).

With this study, we found that upwards lifting of the anterior abdominal wall during abdominal entry provided a mean safe working distance of 3.97±1.5 cm with the use of towel clamps alone, and 4.53±1.59 cm with the use of towel clamps plus manual lifting from the lower abdomen. These results suggest that the recruitment of both towel clamps and manual lifting for anterior abdominal wall lifting to create pneumoperitoneum, the most important component of laparoscopic surgery, provided a significantly greater distance. This distance resulting from maximal lifting might provide a safe working space to laparoscopists to prevent intestinal and vascular injuries.

### Study limitations

The main limitation of our study is the lack of measurement of the traction force of anterior abdominal wall with a standardized device. Although study procedures were performed by the same two surgeons and the same surgeon performed the anterior abdominal wall lifting throughout the study, the possibility of exertion of different traction forces in each case cannot be ruled out.

Our results explicitly demonstrated that manual lifting of the anterior abdominal wall provided significantly greater intra-abdominal distance prior to first entry with a promising protective effect for the closest vital structure. Accordingly, the distance was greatest beneath the exact site of lifting. Consequently, despite the achievement of an adequate entry distance with the use of anterior abdominal wall lifting with towel clamps alone prior to direct trocar entry, the recruitment of manual lifting from the lower abdomen as well as towel clamps provide the surgeon with both a statistically significantly greater entry space and a rigid parietal peritoneum that facilitates entry.

## Figures and Tables

**Table 1:**
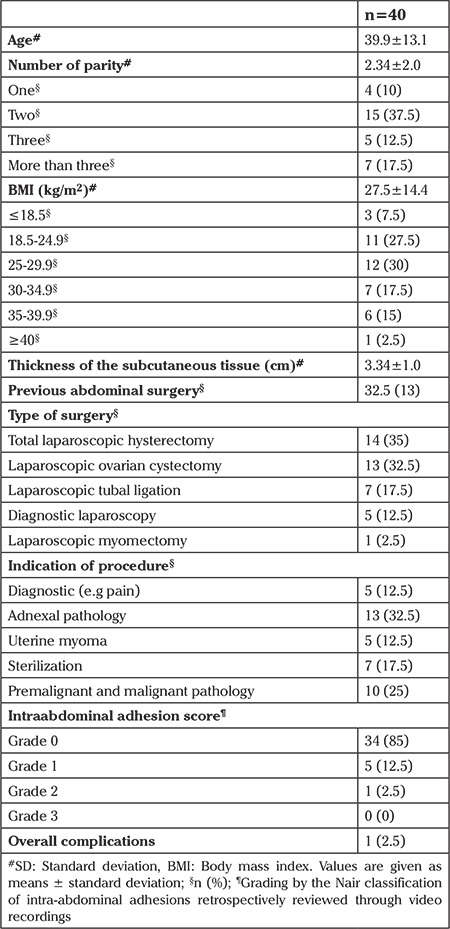
Patient characteristics (all patients)
